# Spontaneous healing of a traumatic critical radius bone defect in adolescent: A rare case report

**DOI:** 10.1016/j.ijscr.2021.105806

**Published:** 2021-03-22

**Authors:** Aryadi Kurniawan, Triadi Wijaya, Witantra Dhamar Hutami

**Affiliations:** Department of Orthopaedics and Traumatology, Cipto Mangunkusumo National Central Hospital and Faculty of Medicine, Universitas Indonesia, Jalan Diponegoro No. 71, Jakarta Pusat, Jakarta 10430, Indonesia

**Keywords:** Critical bone defect, Spontaneous healing, Adolescent bone defect, Rare case, Case report

## Abstract

**Introduction and importance:**

Fracture with a critical bone loss is associated with a profound burden of disease impact. Although there are several options exist for its treatment, but still those reconstructive procedures are technically demanding, relatively expensive and sometimes the result is less than what was expected. The objective of this study is to report a rare case of spontaneous healing of a critical radial bone defect in an adolescent.

**Case presentation:**

We reported a 15 year old boy with a segmental open fracture of left radius, open fracture of left distal shaft ulna and closed fracture of left intercondylar humerus. The middle fragment of a fractured radius was extruded out, pulled out and then thrown away by his parent. Debridement, open reduction, and internal fixation for ulna were performed as well as reposition and internal fixation for the intercondylar humerus fracture. The plan was to wait until the ulnar fracture and intercondylar fracture to heal without any sign of infection and proceed to overcome the radial critical bone defect. This case report had been reported in line with SCARE criteria. The patient showed up seven months later with solid union of the critical radius bone defect and fully functioning hand with only slight limitation in pronation.

**Clinical discussion:**

Osteogenesis in fracture requires osteogenic cells, osteoinductive components, osteoconductive scaffold, and stability. Despite the fact that critical bone defect poses great challenge for its management, intact periosteum and sufficient soft tissue perfusion were able to provide those biologic requirements adequately for fracture healing and ensure spontaneous healing of a traumatic critical bone loss in adolescent without any reconstructive procedure.

**Conclusion:**

Spontaneous healing in critical bone defect is possible, provided all the favorable factors present to support this phenomenon.

## Highlights

• Osteogenesis in fracture requires osteogenic cells, osteoconductive components, and osteoconductive scaffold.
• Intact periosteum and sufficient soft tissue perfusion could be biologically required.
• Pediatric patient with significant bone defect is able to heal the defect speontaneously.

## 1 Introduction

Fractures associated with bone loss is a significant challenge which frequently require repeated surgical interventions. Despite those numerous attempts to overcome bone loss, the results were frequently less than what we hope for. Traumatic bone loss is defined as the expulsion or dissapearance of bone fragment due to trauma or removal of devitalized bone during debridement [1]. General agreement for the definition of critical traumatic bone loss is when the size of the defect is 2–3 times the diameter of the involved bone [2]. Maufrey et al. [3] stated that a critical bone defect generally has circumferential loss >50% or a loss in length of >2 cm. While fracture has its potency for self healing, especially in children and adolescent, the existence of critical bone defect may lead to nonunion due to limitation of musculoskeletal system ability to fill the defects and repair the fracture unless reconstructive bony surgery is performed.

Historically, after several surgical attemps were performed and the result was not functional limb then the extremity was considered mangled and subsequently managed by amputation with a consequence of a considerable loss of quality of life. Nowadays, the epicentrum of management has shifted toward limb salvage procedures which encompasses following options: bone shortening, distraction osteogenesis, the use of vascularized and nonvascularized bone grafts and induced membran techniques [3].

Giannoudis et al. [[4], [5], [6]] stated the *Diamond Concept* as the basic requirement for fracture healing which consist of osteogenic cells, osteoinductive, osteoconductive and mechanical stability. The biological component of such diamond concept were delivered by an intact and adequate soft tissue coverage. Giannoudis et al. [4,6] also stated that acceleration of fracture healing or resolving delayed union and nonunion is possible but it has to pass through adequacy of such biological component of diamond concept and supported with sufficient mechanical stability. Regardless which surgical techniques being used, those reconstructive surgeries to overcome critical bone loss were performed in an effort to deliver biological substance and mechanical fixation required for osteogenesis.

This case report describes a rare case of a spontaneous healing of a critical radial bone defect in an adolescent due to trauma. The aim of this study is to confirm that such spontaneous healing in critical bone defect is possible, provided the periosteum is intact, the soft tissue coverage is adequate and there is no infection. Therefore, it is vital to fulfill those requirements in management of critical bone defect. This case report had been reported in line with SCARE criteria [7].

## 2 Patient information

We reported a 15 year old boy with history of falling from a tree 26 h prior to admission. He fell with his left elbow in full extension and had an open segmental fracture of left radius, open fracture of left distal shaft ulna and closed fracture of left intercondylar humerus. The middle fragment of radius fracture was extruded outward and was then pulled out and thrown away unpurposely by his parent because mistakenly identified the bone fragment as wood that piercing to his son’s forearm. At the time he came to hospital, he didn’t bring the remaining bone fragment.

## 3 Clinical findings

**Fig. 1:**
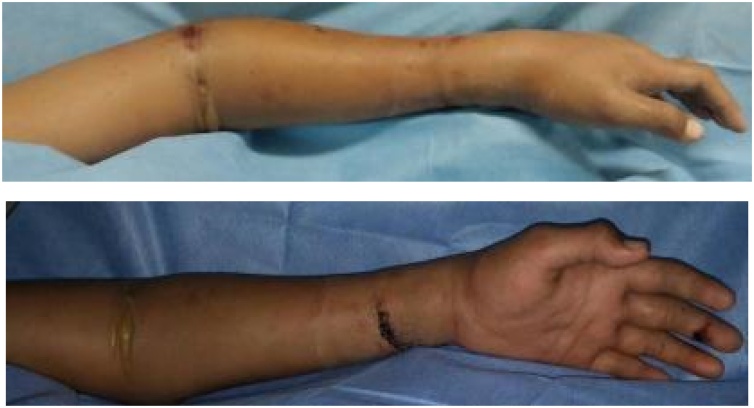
**Clinical Finding during Hospital Admission.** There were swelling and angulation of the left distal forearm, and an open wound.

Physical examination at the time of admission revealed swelling and angulation on the left distal forearm with an open wound size 3 × 0.5 cm. There was also a swelling at the elbow joint with no open wound. Distal perfusion and sensory was normal (Fig. 1).

## 4 Timeline

**Table:** 

Time	Clinical Finding	Treatment
Twenty six hours before hospital admission	Open fracture with bone fragment extruded from the skin and was pulled out	Wound toilet, primary suture of the wound, antibiotic
Twenty six hours after trauma	Pain, swelling, angulation and open wound at the left distal forearm with preserved perfusion and sensory. Motoric was limited due to pain Swelling and pin at the left elbow	Emergency debridement and back slab application
Seven days after admission	Pain, swelling, and angulation at the left distal forearm with preserved perfusion and sensory. Motoric was limited due to pain. Swelling and pin at the left elbow. Wound was left open due to soft tissue swelling	Open reduction internal fixation of ulnar fracture and intercondylar fracture, application of backslab

## 5 Diagnostic assessment

**Fig. 2:**
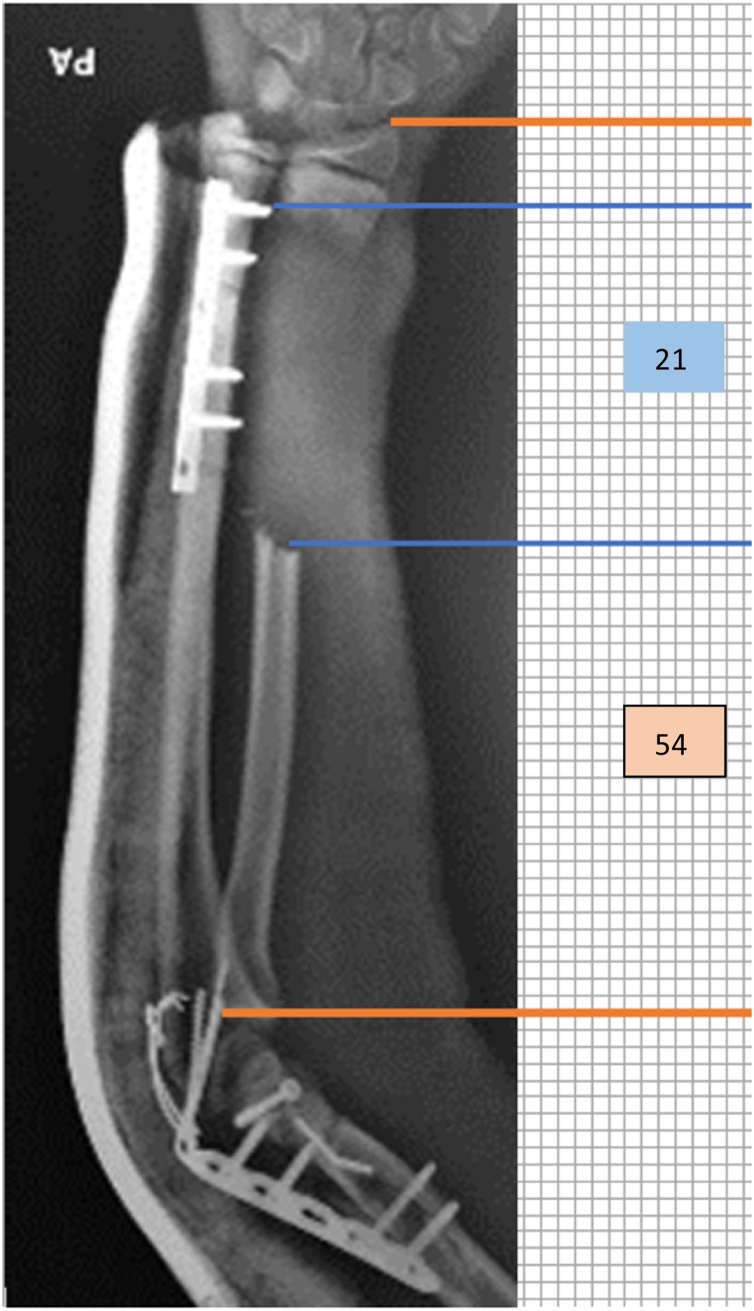
**Bone Defect Measurement.** Gross anatomically, the bone loss was 9 cm length. The percentage of bone loss according to the measurement is 38%, calculated from the 21 boxes loss of bone compared to 54 boxes length of radius.

Anteroposterior and lateral view radiographs of left forearm showed transverse fracture of distal shaft ulna with displacement to side (Fig. 2). There was 9 cm (38%) bone loss ofradius that started from mid shaft radius to metaphysis of distal radius. The elbow’s anteroposterior and lateral view radiographs showed that this patient also got intercondyler humeral fracture with T-shaped fracture line that extending to metaphysis of distal humerus but there was no significant displacement of the fragments.

We diagnosed the patient with open segmental fracture of left radius with bone loss, open fracture of left distal shaft ulna Gustillo-anderson grade II, and closed fracture of left intercondylar humerus Radins Riseborough type II.

## 6 Therapeutic intervention

Two-stage surgeries were carried out, with the emergency debridement and application of back slab performed at emergency operating theatre and the wound was left open due to soft tissue swelling. Second surgery was performed a week after during which open reduction and internal fixation of ulnar fracture and intercondylar humerus fracture were performed, still with the application of backslab postoperatively. The radius bone defect was left untouched to confirm there was no subsequent sign of infection and was planned to have reconstructive surgery after the ulnar and intercondylar fracture healed.

## 7 Follow up and outcomes

**Fig. 3:**
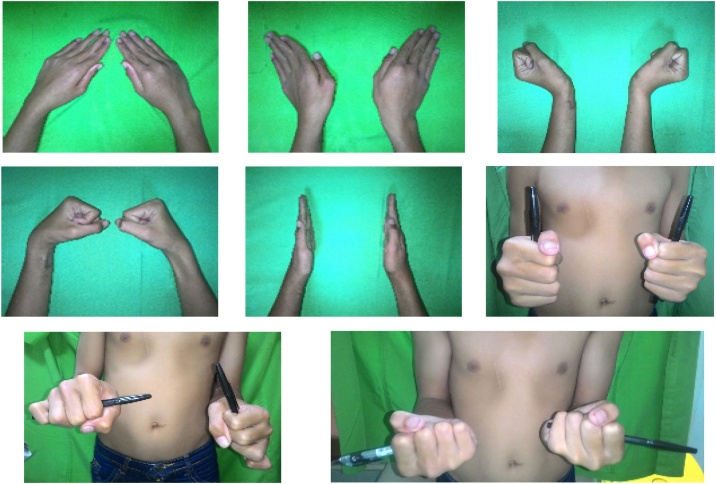
**Seven Months Postoperative Clinical Condition.** Only limited pronantion was found.

Patient attended outpatient clinic for wound care once a week several times, only to stop coming after the wound had healed and came again 7 months afterwards. At 7 months follow up, patient had no pain nor deformity on the forearm and elbow. The range of motion of the wrist and elbow was summarised in Table 1 and shown at Fig. 3.

**Table 1:** Measurement of Wrist Range of Motion Seven Months after Injury.

Variables	Values	Normal Values
**Wrist Profiles**		
Radial height	8 mm	8–17 mm
Ulnar variance	+1 cm	−4 to +2 mm
Radial Inclination	18°	16°–29°
Palmar tilt	28° (dorsal)	0°–22° (palmar)
**Range of Motion of Wrist Joint**		
Flexion	0°–80°	0°–80°
Extension	0°–70°	0°–70°
Pronation	0°–40°	0°–90°
Supination	0°–90°	0°–90°
Ulnar deviation	0°–30°	0°–30°
Radial deviation	0°–20°	0°–20°

**Fig. 4:**
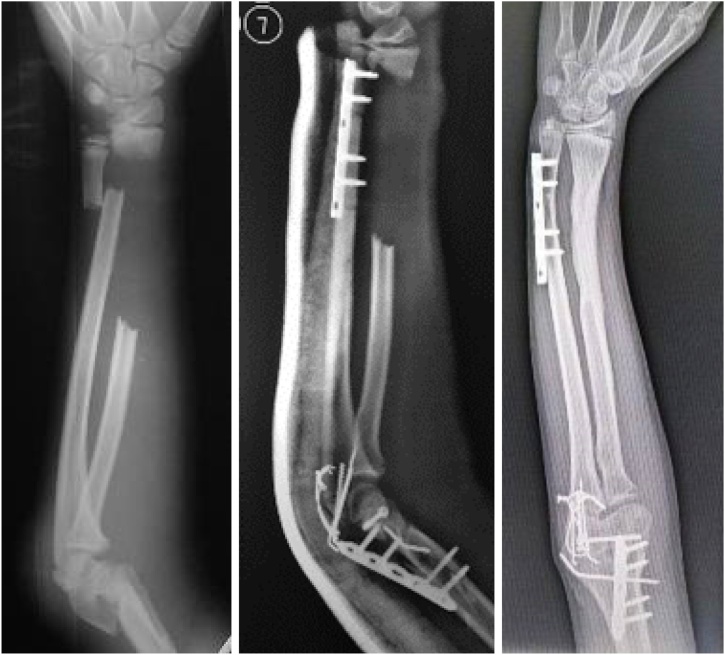
**Sequential X Ray Findings of the Patient.** (Left) at initial encounter, the radial bone defect was measured as 9 cm (38%) bone loss. (Middle) at a week after initial encounter, the ulnar fracture was fixated using plate and screw. (Right) seven months after initial encounter, the bone defect had been filled with new bone formation.

Patient had normal wrist flexion, extension, radial deviation, ulnar deviation, and supination but limited pronation. The 7-months postoperative x ray showed the fracture of ulna and intercondylar humerus had been united and the radius critical bone defect was filled with solid bone with no gross angulation (Fig. 4).

## 8 Discussion

Most patients with traumatic bone loss will come to emergency room as an emergency open fracture case [8]. It is important to carefully assess the perfusion and degree of contamination of the wound so that adequate and proper management can be delivered. The goals of treatment are not only to achieve bony union but also restoration of function as soon as possible. Due to development of bone defect management method and soft tissue reconstruction technique, critical open bone loss was mostly treated with two stage treatment. First stage is meticulous debridement and removal of devitalised tissue followed by reconstruction of soft tissue, if necessary, to ensure sufficient coverage. Debridement itself may create a bone defect or even extend the existing bone and soft tissue defect. Once the soft tissue coverage and infection issues have been fully addressed, the second stage is bone reconstruction surgery to fill in the bone gap which may be one of these option: bone shortening, distraction osteogenesis, the use of vascularized and nonvascularized bone grafts and induced membran techniques [1,3]. Regardless the technique being used, treatment of critical bone loss has to fulfill preservation of limb length and alignment, solid union of bone and restoration of function.

Autogenous bone graft as the golden standard management for bone gap has been successfully treated bone defect less than 5 cm. It provides growth factors, osteogenic cells and allows for early revascularization that eventually leads to a high incorporation rate. The challenge for autogenous bone graft is the limited availability, especially in children, and donor site morbidity. The challenge grows bigger when we are dealing with a large bone defect. Vascularized bone graft can treat a defect until 10–20 cm length [3] but it requires skill demanding microsurgery, long term immobilization, and rehabilitation. Other disadvantages of this microsurgery are possibility of non-union in the docking site, stress fractures of the graft and a lengthy period for graft to grow and reach the desired dimension. Distraction osteogenesis is another option of treatment for bone loss and may sucesfully manage until 10 cm of bone defect [3]. The major drawbacks are this technique is cumbersome for the patients, possibility of recurrent pin tract infection, requirement of additional surgeries for fixator re-alignments and wire re-tensioning. Additional surgeries are also frequently needed for debridement and promote union at the docking site. From the patients side, this treatment requires very good patient compliance due to its long and frequent surgical intervention [1,9].

One major difference between adult and pediatric in bone regeneration is the characteristic of periosteum. Periosteum in pediatric has huge osteogenic capacity which is generated by osteoprogenitor cells in its inner layer, as well as the ability to deliver osteoinductive substances. The presence of periosteal sleeve which bridge the fracture gap is important in spontaneous healing of bone loss. In adults, periosteum is much weaker, thinner and more adherent to the bone while in children the periosteum is thicker and less adherent that it will easily be stripped off from the underlying bone. The less adherent periosteum is one of reason it may stay relatively intact in a bone defect [10]. In the traumatic bone loss with relatively intact periosteum sleeve, the defect will be filled by hematoma and osteogenesis will be initiated by the inner layer of periosteum that initially produce the cartilaginous tissue which later on will ossify [10]. The presence of muscular soft tissue coverage is also utmost important since it ensures perfusion and deliveries of growth factors. The absence of infection is also pivotal because infection may retard the process of fracture healing and since almost all fractures with bone defect started as an open fracture then initial management of open fracture is very important as well. Therefore, conservative treatment for bone defect is actually possible provided all required components for fracture healing is sufficiently available. The big question is what is the maximum length for bone defect which can be managed conservatively?

In our case, patient fell from a tree and got an open segmental fracture of radius with a bone fragment extruding from inside out. The bone loss from in this case was due to his parent pulling the fragment out and not from the initial trauma. There was no major soft tissue compromise and by the mechanism of bone loss, we might assume that the periosteal sleeve was still relatively intact and the surrounding muscle envelope was still relatively undamaged. Debridement and other proper management for open fracture was performed to ensure contamination was fully addressed and infection did not happen. The patient is 15 years of age which can still be considered as within growth spurt of puberty during which the osteogenesis is enhanced due to the presence of sex hormone. The presence of sex hormone during growth spurt may enhance fracture healing [11] Adequate stability was provided by open reduction and internal fixation of ulna supported with back slab for the first few weeks. Early mobilization was also made possible by open reduction and internal fixation of the elbow intercondylar fracture. This patient had all of the favorable factors that support spontaneous healing of the critical radius bone defect and such spontaneous healing may overcome 9 cm bone defect (38%) in a 15 years old boy.

## 9 Patient perspective

Patient and family had been informed regarding the condition, treatment, result of the treatment, and prognosis. Patient and family understood.

## Declaration of Competing Interest

The authors certify that They have NO affiliations with or involvement in any organization or entity with any financial interest or non-financial interest in the subject matter or materials discussed in this manuscript.

## Sources of funding

The authors received no financial support for the research, authorship, and/or publication of this article.

## Ethical approval

The ethical approval was not required for this case report.

## Consent

Written informed consent was obtained from the patient for publication of this case report and accompanying images. A copy of the written consent is available for review by the Editor-in-Chief of this journal on request.

## Author contribution

Aryadi Kurniawan: study concept, data collection, data interpretation, and writing the paper

Triadi Wijaya: data collection, data interpretation and writing the paper

Witantra Dhamar Hutami: data collection, data interpretation and writing the paper

## Registration of research studies

This case report is not a first in man study.

## Guarantor

Aryadi Kurniawan.

## Provenance and peer review

Not commissioned, externally peer-reviewed.
